# The structure, folding kinetics, and dynamics of long poly(UG) RNA

**DOI:** 10.1093/nar/gkaf685

**Published:** 2025-07-19

**Authors:** Riley J Petersen, Rahul Vivek, Marco Tonelli, Saeed Roschdi, Samuel E Butcher

**Affiliations:** Department of Biochemistry, University of Wisconsin-Madison, Madison, WI 53706, United States; Department of Biochemistry, University of Wisconsin-Madison, Madison, WI 53706, United States; Department of Biochemistry, University of Wisconsin-Madison, Madison, WI 53706, United States; National Magnetic Resonance Facility at Madison, University of Wisconsin-Madison, Madison, WI 53706, United States; Department of Biochemistry, University of Wisconsin-Madison, Madison, WI 53706, United States; Department of Biochemistry, University of Wisconsin-Madison, Madison, WI 53706, United States; National Magnetic Resonance Facility at Madison, University of Wisconsin-Madison, Madison, WI 53706, United States

## Abstract

Long poly(UG) or “pUG” dinucleotide repeats are abundant in eukaryotic transcriptomes. Over a thousand human genes have pUGs longer than 24 repeats, including the cancer-associated lncRNA NEAT1. In *C*. *elegans*, enzymatic addition of long pUGs to RNA 3′ ends (pUG tails) marks RNAs as vectors of gene silencing. Gene silencing requires at least one pUG fold, a left-handed quadruplex structure that incorporates 12 repeats, but longer pUG tails are more effective. Here, we investigate the structure, folding kinetics and dynamics of long pUG RNAs. RNAs with 24 or more repeats slowly form compact, double pUG folds. The forward rate of pUG fold formation *in vitro* is length-dependent with a half-life (t_1/2_) of 13 min or more, while the unfolding rate is very slow (t_1/2_ ∼5 days). Long pUG RNAs display biphasic dynamics with an additional, faster unfolding phase (t_1/2_ ∼30 min). The amplitude of the faster phase indicates partial unfolding. From these data we propose a dynamic model for segmental register exchange and double pUG fold formation in long pUG RNAs. These data broaden our understanding of the structure and dynamics of long pUG RNAs and have implications for understanding the roles of pUG folds in biology and disease.

## Graphical Abstract

**Graphical Abstract:**
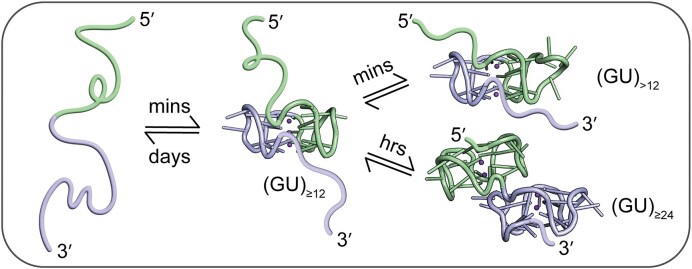

## Introduction

**Figure 1.**
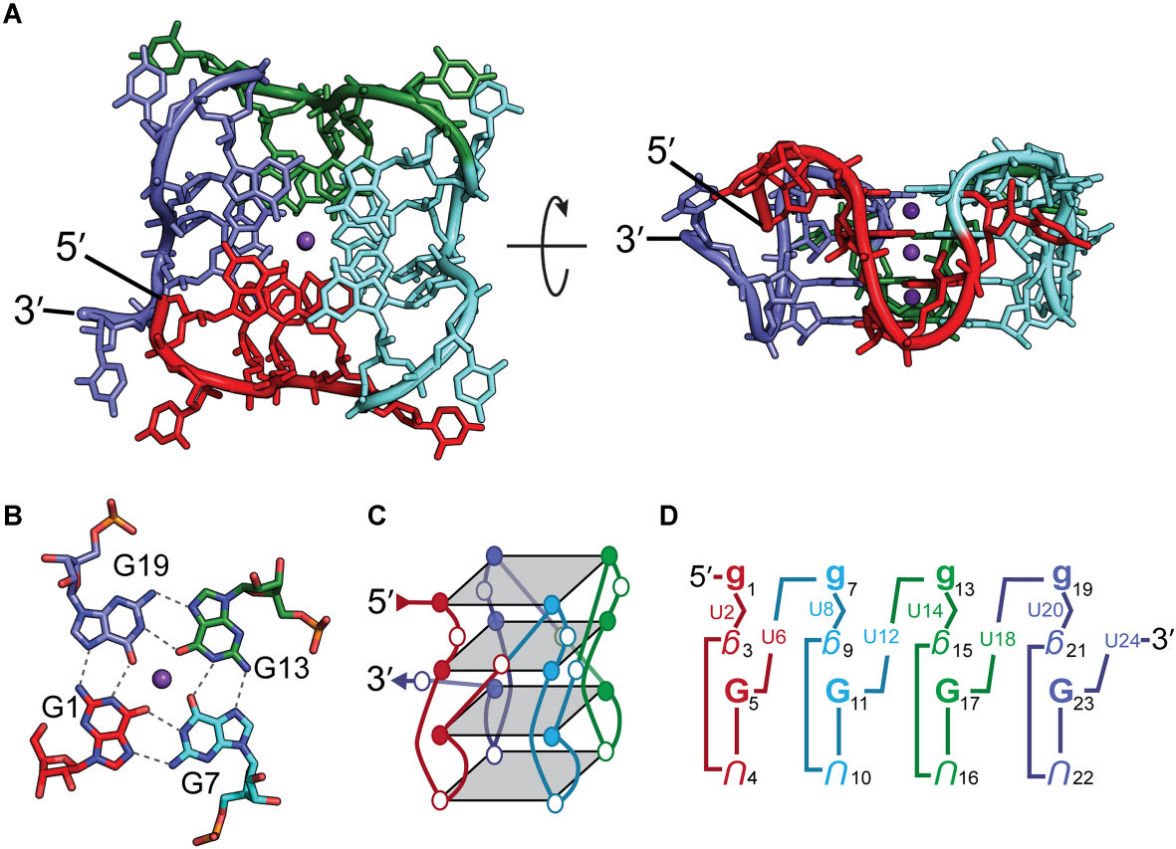
(**A**) Structure of the (GU)_12_ pUG fold (PDB: 8TNS, 7MKT). The left-handed G4 is comprised of four segments, each with 3 GU repeats. The segments are color coded from 5′ to 3′ as red, cyan, green, and blue. Three central potassium ions are shown in purple. (**B**) The G1-quartet. (**C**) Schematic of the pUG fold topology, with G and U nucleotides shown as filled and open circles, respectively. (**D**) Short-hand description of the pUG-fold. Upper case nucleotides are *Anti*, and lower case are *Syn*. Bold nucleotides are 2′-endo and italicized nucleotides are 3′-endo. Inverted nucleotides indicate inverted strand polarity relative to the 5′ nucleotide. Small Us represent bulged or looped nucleotides..

Poly(UG) or pUG RNAs are dinucleotide simple sequence repeats (SSRs) that are abundant in eukaryotes. SSRs make up ∼3% of the human genome [1] and the most common SSR in mammals is GT/AC [2]. Polymorphisms in GT/AC SSRs have been linked to diseases including cystic fibrosis [3, 4], asthma [5], cardiovascular disease [6], schizophrenia [7, 8], and colorectal cancer [9]. GT dinucleotide repeats are more often transcribed than AC repeats, as they tend to be downstream of promoter regions [10]. Thus, eukaryotic transcriptomes are enriched in GU repeats. These repeats can begin with either a G or U and are collectively referred to as poly(UG) or “pUG” RNAs [11]. Previously, we discovered the pUG fold, an unusual left-handed quadruplex (G4) that incorporates 12 UG repeats (Fig. 1) [11–13]. Humans have ∼20,000 pUGs with 12 or more repeats [11]. Among all dinucleotide SSRs with 12 or more repeats in humans, pUGs are the only ones that have a non-random distribution and are enriched near 5′ and 3′ splice sites [11]. Expansion of a pUG repeat in the CFTR gene causes mis-splicing and a rare hereditary form of cystic fibrosis with male infertility [3, 14]. The long noncoding RNA (lncRNA) nuclear paraspeckle assembly transcript 1 (NEAT1) has 29.5 pUG repeats and is overexpressed in many cancers [15–19].

The SSR distribution in *C*. *elegans* is unusual, as pUGs are nearly absent within the encoded transcriptome but instead are post-transcriptionally added to RNA 3′ ends by the enzyme RDE-3 [20, 21]. These “pUG tails” mark RNAs as vectors for gene silencing and can be over 100 nucleotides long [20, 21]. Gene silencing in *C*. *elegans* requires the pUG fold (Fig. 1) [11]. The unique left-handed G4 topology of the pUG fold recruits the RNA-dependent RNA polymerase (RdRP) RRF-1 to RNA 3′ ends for secondary small interfering RNA (siRNA) synthesis [11, 20]. These siRNAs can target additional mRNAs for cleavage and “pUGylation” in a feed-forward amplification cycle [20]. The pUG-tailed mRNAs are packaged in embryos and inherited. A single injection of a pUG tailed RNA into an adult animal promotes gene silencing for six generations, a phenomenon known as transgenerational epigenetic inheritance (TEI) [20]. The pUG-mediated siRNA amplification pathway provides an explanation as to why GT repeats are very rare in the *C*. *elegans* genome [11].

The pUG fold has 3 G quartets, 1 U quartet, single uridine bulges, and single uridine loops [11–13] (Fig. 1). The pUG fold forms around a core of potassium ions and is the only known left-handed RNA G4, owing to a Z-form like backbone configuration in the center of the fold [12]. The pUG fold can tolerate insertion of AA dinucleotides while maintaining its structure and gene silencing activity [11]. Intramolecular pUG folds are not restricted to RNA 3′ ends and can form in the middle of RNAs *in vitro* [11]. The pUG fold requires 12 guanosines [11]. Active pUG tails also minimally require 12 guanosines, but gene silencing activity increases with pUG tail length and plateaus somewhere between 18.5 and 40.5 repeats [11].

In this study, we examine the structure, folding kinetics and dynamics of pUG RNAs as a function of length. Circular dichroism (CD) spectroscopy, nuclease mapping, native gel electrophoresis, nuclear magnetic resonance (NMR) spectroscopy, and molecular dynamics (MD) simulation experiments show that long pUGs with ≥24 repeats can form compact double pUG folds. *In vitro* folding of long pUG RNAs results in a stochastic mixture of single and double pUG folds that slowly resolve into double pUG folds over the course of hours. The forward folding rate of the pUG fold depends on the number of repeats and has a half-life measured in minutes. NMR measurements of hydrogen/deuterium exchange (HDX) rates reveal a slow unfolding process measured in days. Long pUGs have biphasic unfolding kinetics that include an additional faster phase measured in minutes. The amplitude of this faster phase indicates partial but not global unfolding. These data are consistent with a segmental register exchange model involving flanking sequences on either side of a pUG fold. These data improve our understanding of the structure and folding dynamics of long pUGs involved in gene silencing and many other biological processes.

## Materials and methods

### RNA preparation

RNAs were either chemically synthesized by Integrated DNA Technologies or Dharmacon (Horizon) or prepared by *in vitro* transcription with T7 RNA polymerase. Transcribed RNAs used for NMR experiments had 5′-triphosphates removed with calf-intestinal alkaline phosphatase (Invitrogen) as previously described [11] resulting in all NMR samples having 5′ hydroxyls. All RNAs were purified by denaturant PAGE (8–15% polyacrylamide in 7 M urea and TBE buffer) followed by anion exchange chromatography in Hi-trap Q column (Cytiva). Following purification, RNAs were concentrated and exchanged into pure water using Amicon Ultra 3 or 10 kDa spin filters. Buffer components were added, and the RNA was folded via heating and slow cooling in 1 L of 90°C water that was allowed to cool to room temperature over the course of 5–6 h.

### Oligonucleotide sequences

(GU)_12_ : 5′-GUGUGUGUGUGUGUGUGUGUGUGU-3′

(GU)_13_ : 5′-GUGUGUGUGUGUGUGUGUGUGUGUGU-3′

(GU)_15_ : 5′-GUGUGUGUGUGUGUGUGUGUGUGUGUGUGU-3′

(GU)_18_ : 5′-GUGUGUGUGUGUGUGUGUGUGUGUGUGUGUGUGUGU-3′

(GU)_24_ : 5′-GUGUGUGUGUGUGUGUGUGUGUGUGUGUGUGUGUGUGUGUGUGUGU GU-3′

HO-1 pUG: 5′-GUGUGUGUGUGUGUGUGUGUGUGUAUGUGUGUGUGUGUGUGUGUGUGUGU-3′

(GU)_29_ : 5′-GUGUGUGUGUGUGUGUGUGUGUGUGUGUGUGUGUGUGUGUGUGUGUGUGUGUGUGUGU-3′

(GU)_36_ : 5′-GUGUGUGUGUGUGUGUGUGUGUGUGUGUGUGUGUGUGUGUGUGUGUGUGUGUGUGUGUGUGUGUGUGUGUGU-3′

(GU)_50_ : 5′-GUGUGUGUGUGUGUGUGUGUGUGUGUGUGUGUGUGUGUGUGUGUGUGUGUGUGUGUGUGUGUGUGUGUGUGUGUGUGUGUGUGUGUGUGUGUGUGUGUGU-3′

GUAA(GU)_11_ : 5′-GUAAGUGUGUGUGUGUGUGUGUGUGU-3′

(GU)_3_AA(GU)_9_ : 5′-GUGUGUAAGUGUGUGUGUGUGUGUGU-3′

### Circular dichroism

CD was recorded using an AVIV model 420 CD spectrometer using quartz cells with a 1 mm path length. CD samples contained roughly 20 μM RNA in 20 mM potassium phosphate buffer, pH 7.0, 100 mM KCl. CD spectra were recorded at 25°C using a scan step size of 1 nm and 5 s averaging time, measurements were taken from 210 to 340 nm. Raw CD signal was converted to molar CD absorption, Δϵ.


(1) \begin{eqnarray*}
\Delta \varepsilon = \frac{\theta }{{32980 \times C \times L \times N}}
\end{eqnarray*}

where θ is the raw CD signal (millidegrees), C is RNA concentration (M), L is cuvette path length (cm), and N is number of nucleotides. Thermal denaturation studies utilized a temperature ramp from 20°C to 81.5°C in 1.5°C intervals with a 5-min equilibration time at each temperature. Ellipticity was measured at four different wavelengths (243, 264 or 304, 284, and 340 nm), with an averaging time of 5 s at each temperature. Melting temperature, *T*_m_, was determined from the thermal unfolding profile at 243, 264 or 304, and 284 nm by fitting to the Boltzmann sigmoidal equation in Origin (Origin 2020, Origin Lab Corporation).

### pUG folding kinetics

Folding was initiated by manually mixing the RNA with 20 mM BIS-TRIS, pH 7.0 buffer, and 150 mM KCl with or without 2 mM MgCl_2_ to a final concentration of 15–20 μM. Folding kinetics were monitored using CD spectroscopy at four wavelengths corresponding to the characteristic peaks of the pUG fold (243, 264, 284, and 304 nm) and one control (340 nm). Ellipticity was measured for 3–5.5 h with 100–180 data points with 3 s averaging time. Final CD spectra were collected on all samples to ensure proper folding. Raw CD signal was converted to molar CD absorption (Δϵ) via equation 1. Data were measured in duplicate to ensure reproducibility. Forward folding half-lives were determined by fitting the Δϵ versus time curves to a single exponential model using least squares regression in GraphPad Prism. Control experiments containing no salt showed no evidence of pUG fold formation.

### RNase mapping

The (GU)_29_ RNA was prepared in 50 mM Tris, pH 7.0 with 150 mM KCl or 20 mM LiCl with 20 μM RNA. The samples were folded as described above. RNase treatments used 0.1 U/μL or 0.01 U/μL RNase T1 (Thermo Scientific) for 4 min for the KCl sample and 0.001 U/μL RNase T1 for 1 min for the LiCl sample, both at room temperature (∼20°C). Treatments were stopped by the addition of an equal volume of 100% formamide. RNase resistant populations were visualized via SYBR Gold nucleic acid gel stain (Invitrogen) stained denaturant PAGE (10% polyacrylamide in 7 M urea and TBE buffer) and fluorescence imaged on an Amersham Typhoon RGB/5 laser-scanner platform. RNase resistant populations were quantified using ImageJ software.

### Native gel analysis

RNA samples were prepared in 20 mM BIS-TRIS, pH 7.0 with 150 mM KCl or LiCl and contained 20 μM RNA. RNA was loaded with 20% sucrose onto a 1.5 mm thick 10% polyacrylamide native electrophoresis gel containing 5 mM KCl and 1X TBE. The gel was run at 5 W for 2.75–3 h at 4°C with 5 mM KCl in the running buffer. The RNA bands were visualized via staining with toluidine blue.

### NMR spectroscopy

NMR data were collected at the National Magnetic Resonance Facility at Madison on Bruker Avance III HD 600 MHz or 750 MHz and Varian VNMRS DD 800 MHz spectrometers all equipped with cryogenic probes. The temperature of all samples was regulated at 20°C. NMR samples were prepared in 20 mM potassium phosphate buffer, pH 7.0 and 100 mM KCl. After folding 10% D_2_O and 20 μM 4,4-dimethyl-4-silapentane-1-sulfanate (DSS) were added. The same set of experiments were recorded for (GU)_12_, (GU)_13_, (GU)_15_, and (GU)_18_. Two 1D ^1^H spectra were recorded: the first being a full spectrum with water suppression using excitation sculpting with gradients and the second was using a gradient echo with a selective proton pulse centered on the imino protons, referred to henceforth as a 1D SOFAST. The 2D ^1^H,^1^H NOESY spectra collected in H_2_O used a 2D version of a SOFAST-NOESY ^15^N-HMQC experiment [22] optimized for imino protons with a 200 ms mixing time. Samples were lyophilized and transferred to 100% D_2_O and 2D ^1^H,^1^H NOESYs, with 200 ms mixing times were recorded to observe the NOEs between non-exchangeable protons. 1D ^31^P spectra were recorder using power-gated decoupling. 1D spectra were processed using MNova software and chemical shifts were referenced to the DSS chemical shift standard set to 0 ppm. The 2D spectra were processed using NMRPipe [23] and analyzed using NMRFAM-Sparky [24].

### HDX NMR

All HDX experiments were conducted following the same procedure. RNA samples with concentrations between 100 and 250 μΜ were prepared in NMR buffer in 90% H_2_O:10% D_2_O. Pre-exchange 1D ^1^H and 1D SOFAST spectra centered on the imino region were collected. The sample was removed from the NMR tube and lyophilized to remove solvent. HDX experiment is initiated by the rapid addition of 300 μL of 99.99% D_2_O at which point the time was noted and data collection began immediately with the first spectra collected within 15–20 min of initiation. Imino proton signal was observed using 1D SOFAST spectra that were continuously recorded for approximately 3 days with additional spectra recorded over the following days/weeks. All spectra were recorded at 20°C and used the same acquisition parameters. A sample temperature of 20°C was maintained throughout the course of the experiment. Spectra were processed in MNova and peak integrations were recorded. Integral values were normalized relative to the first data point collected. Data were fit using least squares regression in GraphPad Prism to Equation 2 to determine the observed exchange rate, *k*_ex_[25].


(2) \begin{eqnarray*}
I\left( t \right) = \left[ {I\left( 0 \right) - I\left( \infty \right)} \right]{{e}^{ - {{k}_{ex}}t}} + I\left( \infty \right)
\end{eqnarray*}

Where I(t) is peak intensity measured as normalized absolute integrals at exchange time t. I($\infty$) is the intensity of a fully exchanged sample which can be non-zero due to residual H_2_O in the sample, this value was constrained to 0 as no imino proton signal was observed after months in D_2_O indicating the residual H_2_O to be negligible. For (GU)_12_ the well resolved peaks could be fit separately or grouped by quartet with the fit of the quartet being approximately the same as the average fit of the individual peaks (Table 1). For the longer pUGs, where broadened imino signal made integrating single peaks impossible, the entire imino region was integrated together and fit simultaneously and compared to that of (GU)_12_ fit in the same manor (Table 2). To verify the validity of this method for analyzing the data, integrations of imino region were also split in half and fit separately to ensure the global fit agreed with the regional fit.

**Table 1.** (GU)_12_ HDX results

	I(0)	*k*_ex_ (h^−1^)	Half Life (h)	R squared
G9/15	0.99 ± 0.01	0.0054 ± 0.0003	127.4 ± 5.8	0.976
G3/21	0.82 ± 0.02	0.0064 ± 0.0007	108.8 ± 11.5	0.873
G17	0.94 ± 0.01	0.0114 ± 0.0005	60.7 ± 2.5	0.978
G5/11	0.97 ± 0.01	0.0125 ± 0.0004	55.7 ± 1.6	0.991
G23	0.96 ± 0.02	0.0179 ± 0.0007	38.8 ± 1.4	0.981

**Table 2.** pUG HDX results length comparison

	I(0)	*k*_ex,fast_ (h^−1^)	*k*_ex,slow_ (h^−1^)	t_1/2,slow_ (h)	t_1/2,fast_ (h)	Ratio fast (%)	R squared
(GU)_12_	0.92 ± 0.01	-	0.009 ± 0.0004	74.8 ± 3.5	-	-	0.976
(GU)_13_	1.14 ± 0.03	1.34 ± 0.08	0.008 ± 0.0002	92.1 ± 1.8	0.52 ± 0.03	50.2 ± 1.1	0.998
(GU)_15_	1.08 ± 0.04	1.29 ± 0.13	0.005 ± 0.0004	148.6 ± 11.2	0.54 ± 0.05	52.4 ± 1.6	0.986
(GU)_18_	1.05 ± 0.04	1.01 ± 0.12	0.004 ± 0.0003	171.8 ± 12.2	0.69 ± 0.08	50.8 ± 2.0	0.978

### MD simulation

MD simulation was performed with GROMACS v.2023 [26] within the NMRbox resource [27] using the AMBER forcefield with χOL3 modifications [28]. The (GU)_24_ structure was modeled by joining the 5′ and 3′ ends of two copies of the (GU)_12_ model shown in Fig. 1. The (GU)_12_ model was generated from the solution structure of the pUG fold (GU)_12_ (PDB ID: 8TNS) and the crystal structure (PDB ID: 7MKT). The quartet rmsd between these models is 0.6 Å, and the combination of them allows for visualization of the ions and all nucleotides including the 3′ terminal U24, which is not observed in the crystal structure due to disorder. The (GU)_24_ RNA model was solvated in a cubic box of TIP3P water molecules, with a minimum distance of 1 nm from the RNA to the box edges. K^+^ ions were added to a final concentration of 140 mM to neutralize the system. Energy minimization was performed over 50 000 steps using the steepest descent algorithm. Next, 100 ps of NVT (number of particles, volume, and temperature) equilibration was applied over 50 000 steps using 2 fs timesteps and a modified Berendsen thermostat that gradually increased temperature from 0 to 300 K. This was followed by 100 ps of NPT (number of particles, pressure, and temperature) equilibration using 50 000 steps and 2 fs timesteps with a modified Berendsen barostat with pressure maintained at 1 bar. Long-range electrostatic interactions were calculated using the Particle Mesh Ewald method. A production run of 3000 ns was performed with 2 fs timesteps, and trajectory coordinates were saved every 10 ps. Trajectory analysis was carried out using the GROMACS, and visualized with PyMOL v3.1.1 Schrödinger, LLC.

## Results

### Long pUG sequences contain multiple pUG folds

**Figure 2.**
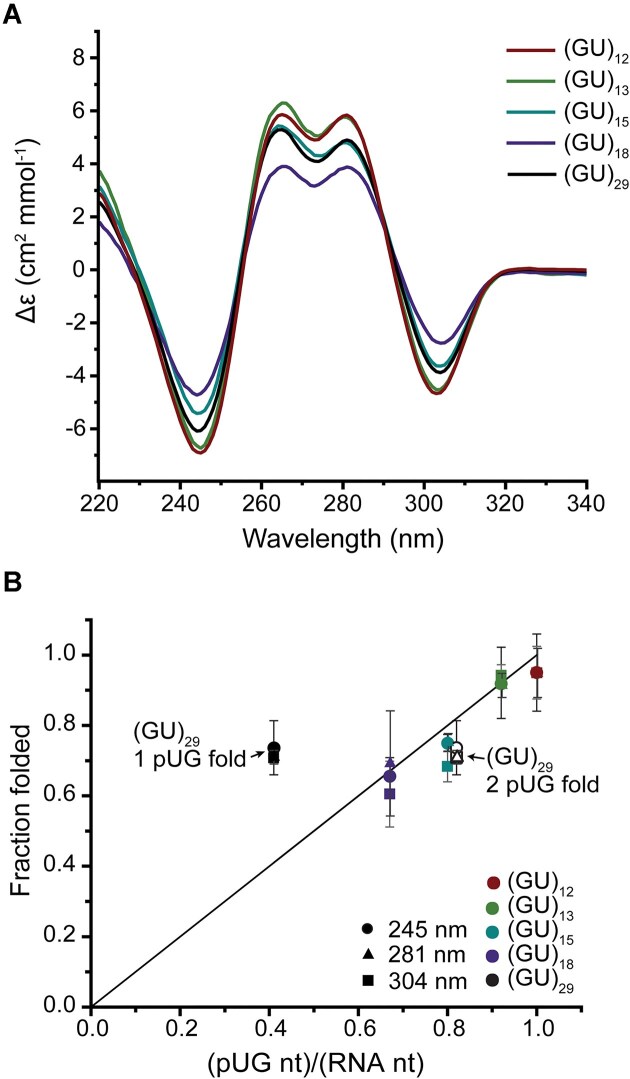
(**A**) CD spectra of (GU)_12_, (GU)_13_, (GU)_15_, (GU)_18_, and (GU)_29_. (**B**) Plot of fraction folded vs nucleotides in a pUG fold per total nucleotides, (pUG nt)/(RNA nt). The fraction folded is measured as the normalized magnitude of the CD signals relative to (GU)_12_, (Δϵ(RNA)/Δϵ((GU)_12_)*0.95) at 245 nm (circle), 281 nm (triangle), and 304 nm (square). Two points for (GU)_29_ depict a folding ratio for 1 pUG fold (filled markers) or 2 pUG folds (open markers).

Humans have > 20 000 pUGs with 12 or more repeats (24 nucleotides), and among these the average length is 17 repeats and the maximum length is 61 repeats (Supplemental Fig. 1). There are > 1500 pUGs sequences with 24 or more repeats in human genes (Supplemental Fig. 1). In *C*. *elegans*, long pUG tails > 18.5 repeats are more effective for gene silencing [11, 20]. We, therefore, investigated the structure and folding of long pUG RNAs. The pUG fold is a surprisingly complex structure despite its simple sequence, with four different layers of quartets, each in a different conformation with respect to sugar pucker, glycosidic bond angle and backbone orientation (Fig. 1). These quartets are: G1-7-13-19, G3-9-15-21, U4-U10-U16-U22, and G5-11-17-23 and hereafter are referred to as the G1, G3, U4, and G5 quartets respectively (Fig. 1). The CD spectrum of the pUG fold is rich in structural information and reports the conformation of stacked nucleobases and the handedness of the backbone [11]. The pUG fold CD spectrum has four peaks (Fig. 2), which can be attributed to the different types of stacking and backbone orientations in the structure. The negative 245 nm peak and positive 260 nm peaks are representative of right-handed parallel *anti-anti* stacking interactions observed in many G4s [29]. The right-handed *anti-anti* interactions in the pUG fold occur at the U4-G5 quartets (Fig. 1D). The 260 nm peak is not unique to G4s and is also a feature of right-handed *anti-anti* stacking in A-form RNA [30]. The negative peak at 304 nm is unusual and reports on the left-handed Z-form *syn-anti* stacking [30, 31] observed at the G3-G5 quartets. Finally, we can attribute the remaining positive peak at 281 nm to the unusual *syn-syn* stacking interactions of the G1-G3 quartets.

We used CD to investigate the folded state of pUG RNAs of various lengths by heating them to 90°C in 130 mM K^+^ buffer and allowing them to slowly cool over the course of 5 h. The CD spectra reveal they all adopt pUG folds (Fig. 2A). The molar CD absorption (Δϵ) also reports the fraction of the RNA chain that adopts the pUG fold. We analyzed the CD peaks at 245, 281, and 304 nm for pUG RNAs of different lengths, which reveals a near-perfect linear trend (*R*^2^= 0.99) for fraction folded versus length (Fig. 2). The minimal pUG fold (GU)_12_ (Fig. 1) displays the greatest molar CD absorption, as all 24 nucleotides are engaged in the pUG fold except for the unstructured 3′ terminal U24 [11] (Fig. 2A). As the chain lengthens, the molar CD absorption decreases as the fraction of folded nucleotides decreases (Fig. 2B). This linear trend indicates an increasing amount of single-stranded RNA (ssRNA) content as the chain grows from 12 to 18 repeats. ^31^P NMR spectra of these RNAs also show the same increase in ssRNA content, observed as a peak of increasing intensity at −0.75 ppm (Supplemental Fig. 2). From integration of the ^31^P NMR data, we can normalize the fraction folded for (GU)_12_ to 95%. Using the normalized molar CD absorption, we can determine the fraction folded of all RNAs; for example, (GU)_18_ is 65% folded and 35% unstructured. The fraction folded agrees well with one pUG fold per RNA for all pUGs ranging from 12 to 18 repeats (Fig. 2B). The 58-nucleotide pUG RNA (GU)_29_ deviates markedly from this linear relationship. (GU)_29_ is long enough to adopt 2 pUG folds and indeed, accounting for 2 pUG folds fits the observed linear relationship of fraction folded (Fig. 2B). Two pUG folds in (GU)_29_ would engage 24 repeats (48 out of 58 nucleotides), corresponding to 83% folded, while a single pUG fold would correspond to only 41% folded. From the measured molar CD absorption, (GU)_29_ is ∼73% folded, corresponding to an average of ∼1.76 pUG folds per 58 nucleotides. Therefore, the majority of the (GU)_29_ RNAs have 2 pUG folds, but some have only one.

**Figure 3.**
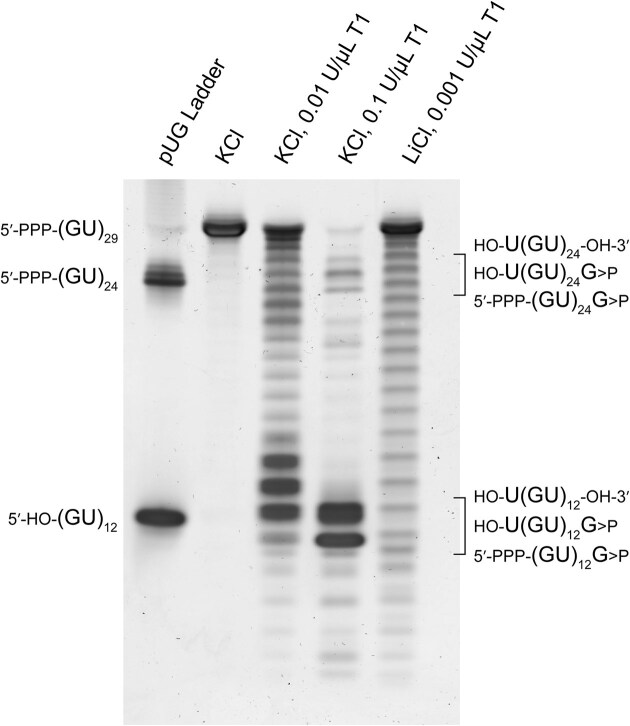
Nuclease mapping of (GU)_29_ using RNase T1. The first lane contains markers for (GU)_12_ and (GU)_24_ RNAs, while the second lane contains untreated (GU)_29_ in K^+^ buffer. RNase treatments are indicated. The three possible RNase resistant fragments are indicated and correspond to 5′ hydroxyl (HO-), 5′ triphosphate (PPP), 3′ hydroxyl, and 3′ cyclic phosphate (>P) termini containing cleavage products.

To further test the idea that multiple pUG folds can form in long pUG RNAs, we subjected (GU)_29_ to extensive degradation with RNase T1, which cuts after single stranded guanosines, and observed the degradation patterns after staining with SYBR-gold nucleic acid stain (Fig. 3). After complete digestion of the intact RNA, we observe RNase-resistant fragments that correspond to single and double pUG folds with 12 and 24 repeats, respectively (Fig. 3). In this experiment, RNAs with one pUG fold or two pUG folds separated by unstructured nucleotides are digested into single pUG folds, while compact, adjacent double pUG folds with no intervening nucleotides remain inaccessible to RNase T1. The electrophoretic mobilities of the cleavage products differ slightly depending upon their termini and the position of the pUG fold in the sequence (for example, a pUG fold at the 5′ end has a 5′ guanosine triphosphate, while pUG folds derived from the middle of the RNA have 5′ hydroxy uridines). The adjacent double pUG fold fragments account for ∼18% of the observed cleavage products. This correlates well with the expected distribution for a stochastic folding model, which predicts 22% of all possible folds should result in adjacent double pUG folds (Supplemental Fig. 3). The stochastic positioning *in vitro* is consistent with previous chemical probing results of single pUG folds in short RNAs [32].

**Figure 4.**
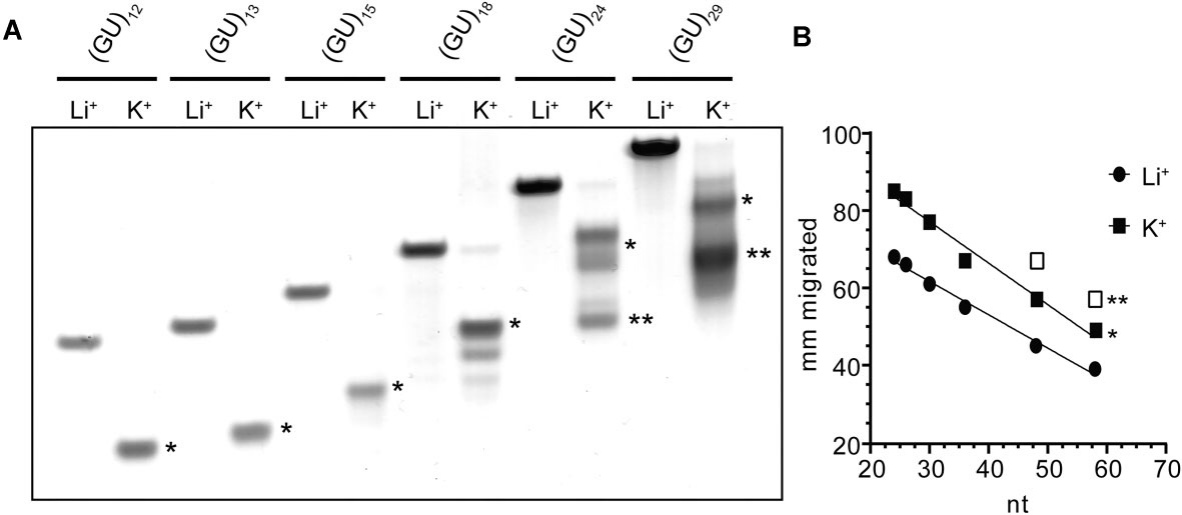
(**A**) Native PAGE analysis of pUG RNAs of various lengths in K^+^ or Li^+^. (**B**) Plot of distance migrated in mm versus the number of nucleotides showing linear relationship (*R*^2^= 0.98) for single stranded pUG RNAs (Li^+^) and single pUG fold bands (K^+^). Double pUG fold outliers differentiated with open squares. (A/B) Single pUG folds and double pUG folds are marked with one or two asterisks, respectively.

**Figure 5.**
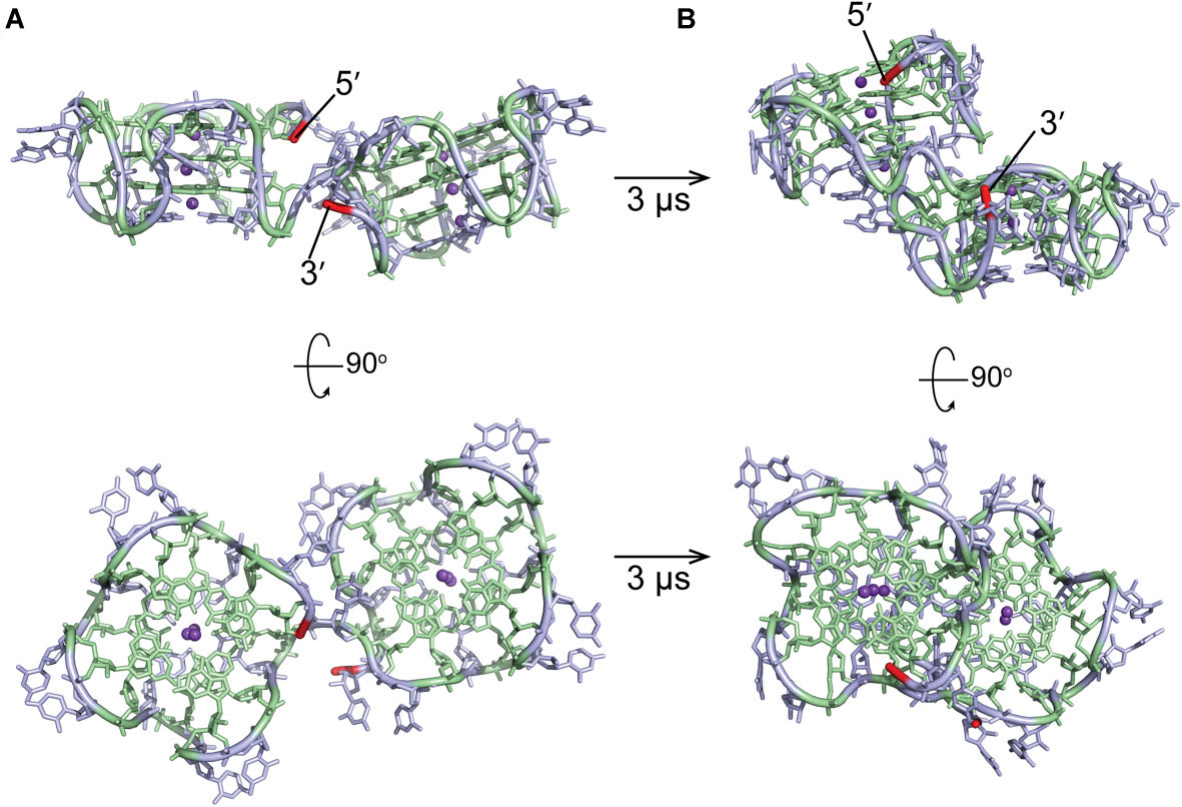
Double pUG fold model of (GU)_24_. Gs are green, Us are blue, and potassium ions are purple. 5′ and 3′ ends are highlighted in red. (**A**) depicts the initial conformation following energy minimization and (**B**) depicts the stacked conformation observed from 486 ns to 3 μs of MD simulation.

We analyzed the conformations of different lengths of pUG RNAs by native PAGE. The pUG RNAs with 12–18 repeats all adopt single pUG folds (Fig. 4). For longer pUG RNAs, the single pUG folds have heterogenous electrophoretic mobilities, which is likely reflective of their different positions within the RNA. The longer pUGs (GU)_24_ and (GU)_29_ both adopt single and double pUG folds (Fig. 4). The assignment of the (GU)_12_ band to a single pUG fold is based on previous data showing it is an intramolecular fold with concentration-independent electrophoretic mobility, as well as previous CD, x-ray and NMR data [11, 13]. Relative to (GU)_12_, we can assign the single pUG folds in longer pUG RNAs by the observed linear trend in electrophoretic mobilities (Fig. 4B). The major band in the longer pUG RNA (GU)_29_ deviates from this linear trend, which is in agreement with CD data indicating the majority of this RNA adopts a double pUG fold. The double pUG folds in (GU)_24_ and (GU)_29_ follow a faster electrophoretic mobility trend (Fig. 4B), consistent with the assignment of double pUG folds in these RNAs. Our previously determined X-ray and NMR structures of the single pUG fold show the 5′ and 3′ ends are closely positioned, but the 3′ terminal uridine is flexible [11, 13]. Nevertheless, it is not immediately apparent how adjacent double pUG folds form and avoid steric clash. We therefore used our previously determined structures [11, 13] to model the adjacent double pUG fold conformation, which we analyzed by MD simulation for 3 μs (Fig. 5). While the initial model was in a side-by-side configuration, a stable, stacked conformation formed after 485 ns and remained stable for the rest of the 3 μs simulation. The stacked conformation results in an increased distance between the 5′ and 3′ ends of the double pUG fold, which may allow additional “beads on a string” pUG fold formation (Fig. 5). Native PAGE analysis of (GU)_36_ and (GU)_50_ are consistent with double and triple pUG fold conformations (Supplemental Fig. 4). Collectively, these data indicate that multiple compact pUG folds can form within a long pUG RNA.

### The kinetics of pUG folding

The folding kinetics of pUG RNAs ranging from 12 to 18 repeats were measured at 25°C using CD following addition of folding buffer with 150 mM K^+^ or 150 mM K^+^ and 2 mM Mg^2+^. All RNA samples contained 15 or 20 μM RNA. The RNAs fold with single exponential kinetics, indicating a monophasic transition from an unfolded to a folded state (Supplemental Fig. 5). In 150 mM K^+^ buffer, the folding half-life of (GU)_12_ is 17 min, (GU)_15_ is 13 min, and the longer pUG RNA (GU)_18_ has the slowest folding rate with a half-life of 30 min. The slow folding of the pUG fold is likely a result of its complex structure which includes 8 backbone inversions and 8 *syn* nucleotide conformations (Fig. 1D). The kinetics of double pUG folding were not measured but are likely even slower. One day after a 5-h folding protocol of heating and slow cooling, 78% of (GU)_29_ contained 2 pUG folds (Fig. 2B). After storage for an additional 1 month at 4°C, 98% of (GU)_29_ contained 2 pUG folds (Supplemental Fig. 6). Thus, long pUG RNAs slowly resolve into double pUG folds over the course of hours to days *in vitro*.

Although Mg^2+^ is not required for the pUG fold, we observe that addition of 2 mM Mg^2+^ increases the rate of folding of the minimal pUG fold (GU)_12_, decreasing the half-life from 17 to 13 min (Supplemental Fig. 5). On the other hand, the folding of longer pUGs is inhibited by the addition of Mg^2+^, as (GU)_15_ folds with a half-life of 36 min in K^+^ and Mg^2+^ buffer and the folding of (GU)_18_ was incomplete after 5 h in K^+^ and Mg^2+^ buffer. The CD spectra of (GU)_18_ in K^+^ and Mg^2+^ buffer shows a positive peak at 260 nm, but the pUG fold peaks at 243, 284, and 304 nm are of much lower intensity (Supplemental Fig. 7). These data are consistent with an A-form helical conformation involving GU wobble pairs competing with pUG folding in longer RNAs. Intermolecular association of long pUGs may be facilitated by the relatively high concentrations (20 μm) needed for the CD measurements, as intermolecular A-form helices are stabilized by Mg^2+^ and tandem GU wobble pairs create K^+^ binding sites [33]. Nevertheless, (GU)_18_ in K^+^ and Mg^2+^ buffer still adopts the pUG fold following a heat and slow cool cycle (Supplemental Fig. 7).

**Figure 6.**
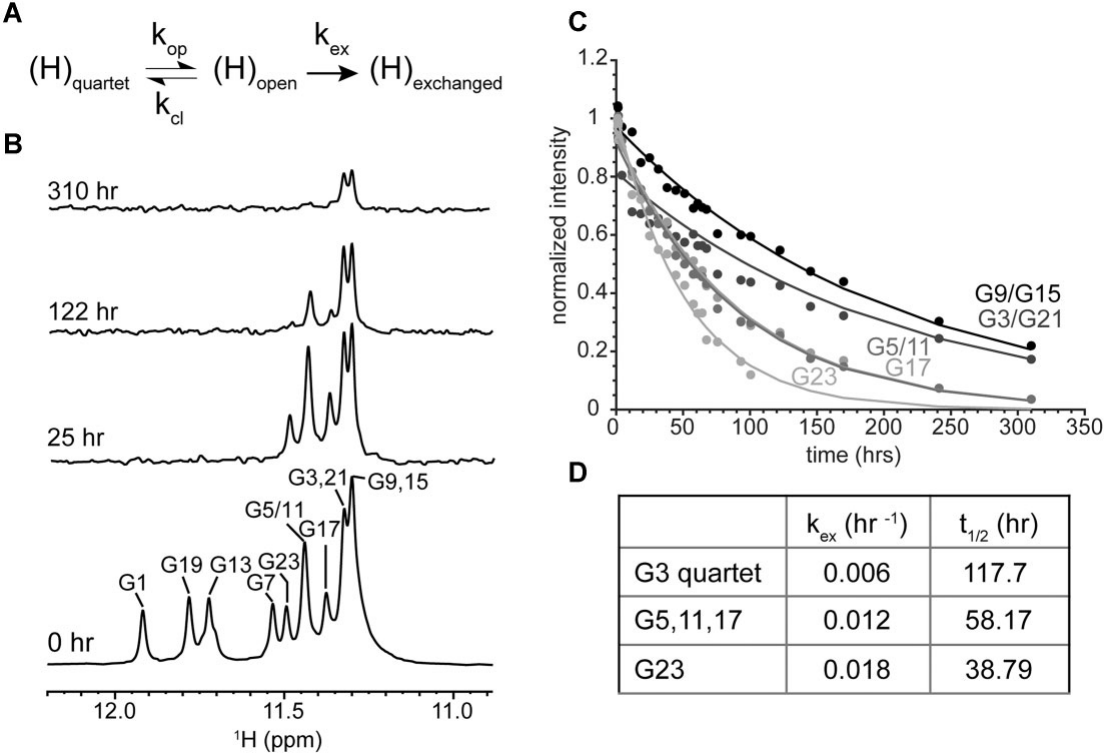
HDX NMR of (GU)_12_. (**A**) HDX mechanism where H-bonded protons in quartets, (H)_quartet_, must open, (H)_open_, to allow the proton to be exchanged, (H)_exchanged_, with deuterium. (**B**) 1D ^1^H NMR showing loss of imino proton signal at given times following transfer into D_2_O. (**C**) Exponential decay of the normalized peak integrals (I(t)/I(0)), over time. (**D**) Summary of average HDX rates (k_ex_) and quartet half-lives for the G3 and G5 quartets, G23 was measured separately.

**Figure 7.**
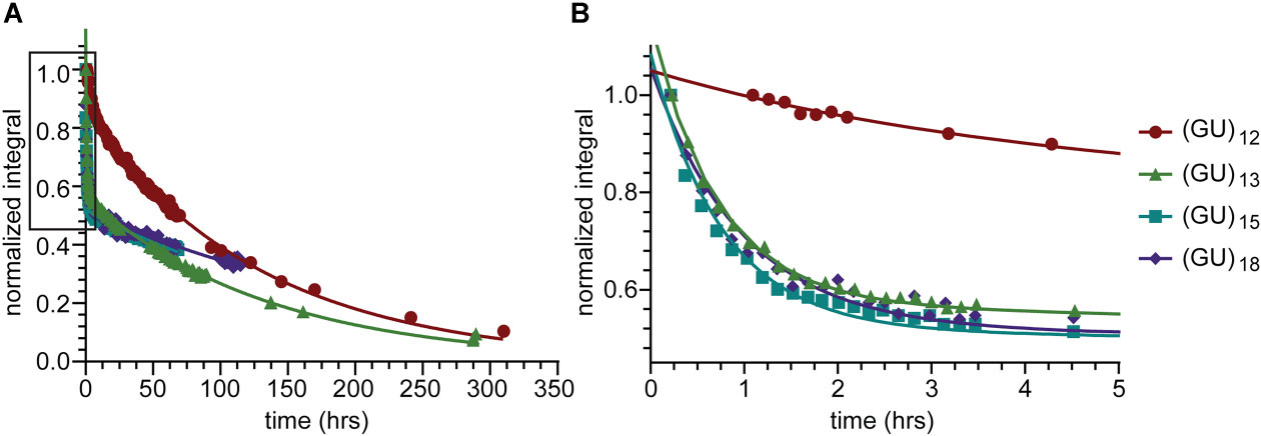
Biphasic HDX of (GU)_13_, (GU)_15_, and (GU)_18_ compared to (GU)_12_. (**A**) Plot of loss of imino proton signal measured as normalized integrals (I(t)/I(0)) of the entire imino region over time fit to an exponential decay for (GU)_12_ and biexponential decays for (GU)_13_, (GU)_15_, and (GU)_18_. (**B**) Zoomed in plot of boxed region from (**A**) highlighting the fast phase of HDX biexponential decay for (GU)_13_, (GU)_15_, and (GU)_18_.

We allowed pUG RNAs to completely fold in K^+^ buffer and measured the kinetics of quartet opening rates and pUG unfolding using HDX NMR. Upon deuterium solvent exposure, the hydrogen bonded imino proton signals disappear over time (Fig. 6)[25, 34–36]. The solvent exposed G1 and U4 quartets exchange rapidly and were not measured, but the imino signals from the buried G3 and G5 quartets are observable for days in D_2_O (Fig. 6B). By fitting the exponential decay of the imino proton signals over time (Fig. 6C), we determined the exchange rates (k_ex_) for the G3 and G5 quartets (Fig. 6D, Table 1), which have half-lives of 5 and 2.5 days, respectively. The slow exchange rate of central quartets may inform on the overall G4 unfolding rate [37]. The G23 guanosine is adjacent to the 3′ end and has a slightly faster exchange rate than the other Gs, which can be attributed to increased dynamics at the more flexible 3′ end [13]. The imino proton signals of (GU)_13_, (GU)_15_, and (GU)_18_ were also observable for days after transfer into D_2_O (Supplemental Fig. 8). However, the longer pUGs exhibit biphasic HDX rates (Fig. 7) with slow and fast exchange processes (*k*_ex,slow_ and *k*_ex,fast_) (Table 2). The slow phase is consistent with the slow quartet opening observed in (GU)_12_ with half-lives in the range of 3.8-7 days (Table 2). The faster exchange process corresponds to half-lives of approximately 30 min. The amplitude of the fast phase is ∼50%, which is consistent with partial but not complete unfolding. Decreased resolution in the NMR spectra of RNAs longer than 12 repeats made fitting of the individual imino peaks not possible. For all RNAs, fits of regions of the spectra were compared to global fits to ensure an accurate measure of pUG fold conformational exchange (Supplemental Fig. 9, Supplemental Table 1).

We hypothesized that pUGs with more than 12 repeats may be able to form pUG folds that incorporate longer loops. Consistent with this, pUGs with AA insertions are still able to induce gene silencing [11]. To further test this looping hypothesis, we enforced loop formation by generating constructs with AA insertions either after U2 or U6, referred to as GUAA(GU)_11_ and (GU)_3_AA(GU)_9_, respectively. Both constructs form pUG folds, as determined by CD (Supplemental Fig. 10). The looped pUG folds have significantly decreased stabilities with melting temperatures of 33.1°C and 40.8°C, respectively (Supplemental Fig. 11).

**Figure 8.**
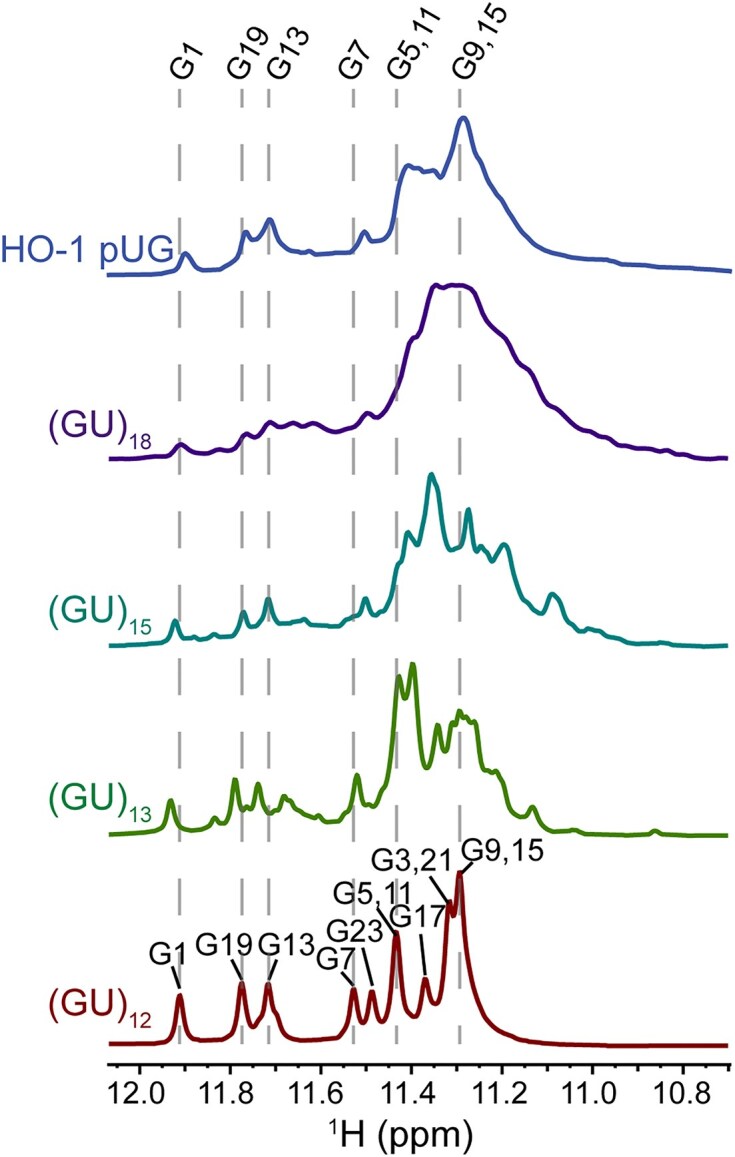
1D ^1^H NMR of (GU)_12_, (GU)_13_, (GU)_15,_ (GU)_18_, and the 50 nt sequence (GU)_12_AU(GU)_12_ from the HO-1 gene. Dotted lines mark chemical shifts of the (GU)_12_ pUG fold.

An increase in line broadening of NMR spectra is observed as pUG length increases, which is indicative of dynamics on the microsecond to millisecond timescale (Fig. 8 and Supplemental Fig. 12). The increase in line broadening is not due to the size of the RNAs, as a larger 50 nucleotide RNA sequence (GU)_12_AU(GU)_12_ derived from the 5′ untranslated region of the human gene heme oxygenase 1 (HO-1) [38] has narrower linewidths than the 36 nucleotide (GU)_18_ (Fig. 8). Despite the line broadening, the pUG fold can be identified in 1D (Fig. 8) and 2D ^1^H,^1^H NOESY spectra for all RNAs (Supplemental Fig. 12). Finally, longer pUG folds also have slightly lower thermal stabilities. The minimal (GU)_12_ pUG fold has a melting temperature of 51.5 °C [11], while (GU)_13_, (GU)_15_ and (GU)_18_ have melting temperatures of 48.3 °C, 47.1 °C and 47.9 °C, respectively (Supplemental Fig. 13). Collectively, our data show that long pUG RNAs form dynamic pUG folds that persist for days.

## Discussion

Here we show that long pUG RNAs with 24 or more repeats can form compact double pUG folds. This is the first experimental evidence for adjacent double pUG folds. Such long pUG repeats are found in thousands of human RNAs. A well-studied example is the lncRNA NEAT1, which has 29.5 pUG repeats. Dysregulation of NEAT1 has been found in almost all cancers and is associated with chemotherapy resistance and poor prognosis [15–18]. The human chromatin modifying enzyme DNMT1 binds to, and is inhibited by, pUG folds [39], and also interacts with NEAT1 [40, 41]. These data suggest pUG folds can form in RNAs with long pUGs and may contribute to disease. Many human pUG repeats are polymorphic with expansions that correlate with higher incidence of disease. For example, a polymorphic pUG is found in the 5′ untranslated region (UTR) of heme oxygenase 1 (HO-1). HO-1 is the enzyme responsible for breaking down heme and must be tightly regulated within a certain cytoprotective range [38]. The pUG repeats in HO-1 are bimodal with short and long isoforms centered on 23 and 30 repeats, respectively [42]. The long repeat isoform (≥25 repeats) has been shown to correlate to higher rates of several diseases such as cardiovascular disease, sickle cell anemia, rheumatoid arthritis and emphysema [6, 43–45]. Repeat length has also been shown to be an independent prognosticator for several cancers, including esophageal and pancreatic [46, 47]. The long repeat isoform is associated with the use of an alternative first exon which contains an alternative transcription start site leading to lower overall expression [38]. HO-1 expression is stimulated by hemin, but hemin also induces expression of 5′ elongated transcripts [38]. Our data suggests that the formation of a second pUG fold in the long repeat isoform may contribute to the mis-splicing of HO-1 or interfere with hemin induced upregulation. Intriguingly, we have previously shown that the pUG fold binds hemin, opening a possible avenue in which HO-1 mRNA may be able to sense hemin to upregulate expression [11].

**Figure 9.**
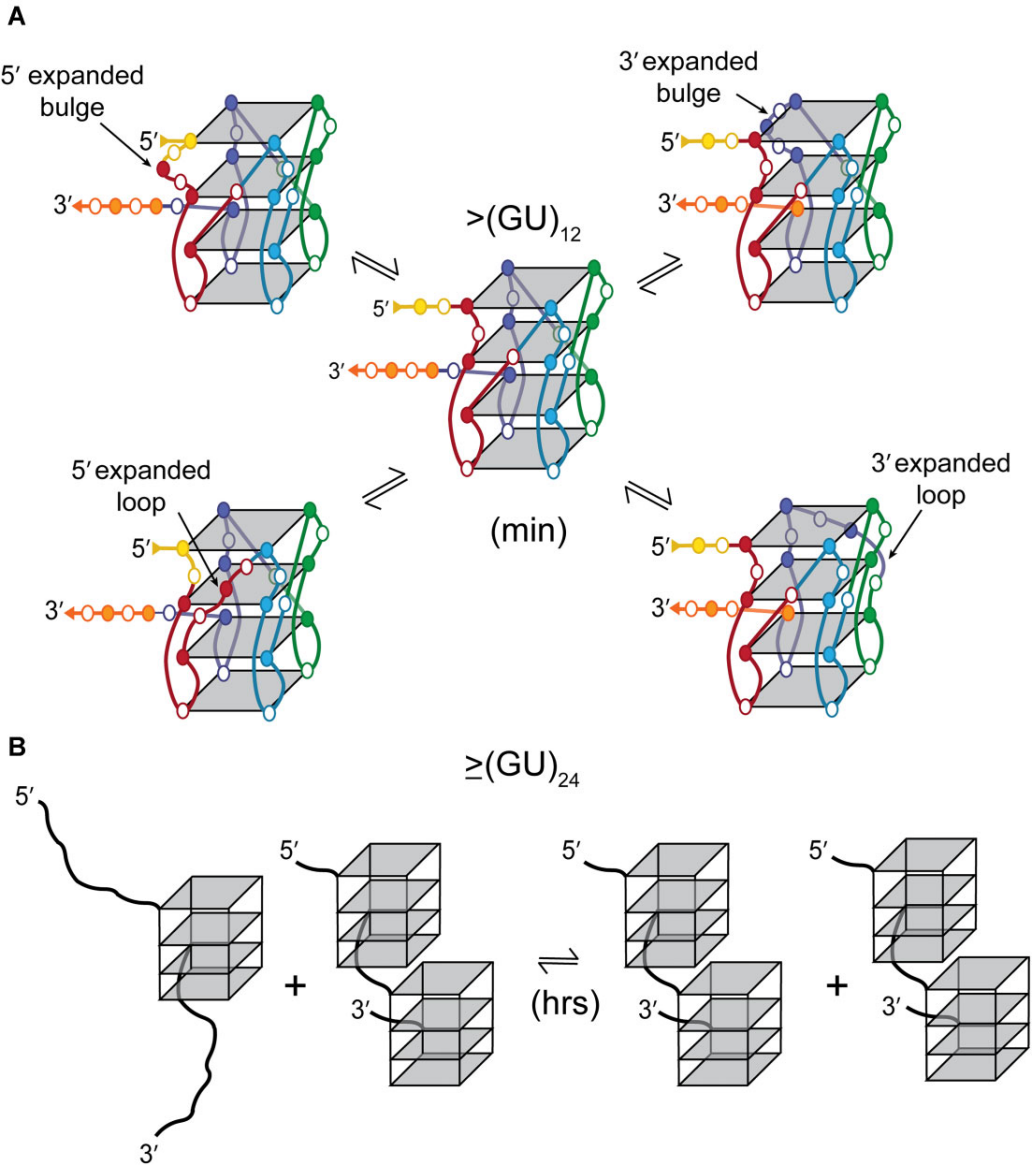
Schematic models of pUG fold dynamics. (**A**) Segmental register exchange of an internal pUG fold in (GU)_15_ involving flanking 5′ and 3′ segments. Gs shown in filled circles and Us shown as open circles. Single stranded nucleotides (yellow and orange) exchange into the structure through the formation of expanded bulges or propeller loops. (**B**) Global structural rearrangement of (GU)_29_ resulting in an increase in double pUG folds.

Although the initial formation of pUG folds *in vitro* is stochastic with respect to their position in the RNA, long pUGs slowly resolve into double pUG folds (Fig. 9B). Folding *in vivo* may be facilitated by co-transcriptional folding and/or pUG RNA binding proteins. The ability to form double pUG folds provides a potential explanation as to why longer pUG tails are more effective at gene silencing *in vivo* [11]. The 3′ end of the pUG fold is flexible, as shown by NMR [13] and the MD simulations reported here and allows the formation of closely packed multimeric pUG folds, like beads on a string. Consistent with the idea of multimeric pUG interactions, pUG repeats are more efficient at phase separation than other G-rich sequences [48]. Stacking interactions between pUG folds likely contribute to condensation and phase separation [48].

The folding and unfolding kinetics reported here are slower than many RNA tertiary structures [49] and other G4s. For example, canonical G4s have forward folding rates of ∼0.1 s^−1^ [50] or faster [51]. In contrast, the forward folding of the pUG fold has a half-life of at least 13 min (Supplemental Fig. 5), corresponding to a rate of 0.0009 s^−1^, which is at least 2 orders of magnitude slower than canonical G4s. The slower forward folding rate may be due in part to the many backbone inversions required for the Z-form like fold (Fig. 1). The pUG fold is also slower to unfold than canonical G4s. The *c-myc* DNA G4 has an average lifetime of ∼2 days [35, 36] while the pUG fold is ∼7 days. The slower unfolding rate may be because the pUG fold has four quartets coordinated to three potassium ions, while canonical G4s typically have three quartets coordinated to two potassium ions [35, 50]. For quadruplexes, the unfolded state does not necessarily imply single-stranded conformations. Recent experiments on quadruplexes in LiCl show unfolded states are more likely dynamic ensembles of hairpin structures [52]. The imino protons of such conformations exchange very rapidly, on the ms timescale [53, 54]. While UG/GU wobble pairs are not nearly as stable as Watson–Crick pairs [12], the unfolded state of pUGs could involve transient formation of hairpins.

Our HDX data reveal that pUG RNAs with more than 12 repeats display an additional dynamic phase that is much faster than unfolding. The amplitude of this faster phase is ∼50%, consistent with partial unfolding. A model depicting such a semi-conservative conformational exchange process is illustrated (Fig. 9A). This model involves the invasion of flanking sequences to induce local register exchange of the 5′ and 3′ segments, accompanied by the formation of expanded loops or bulges (Fig. 9A). This exchange model is similar to the “spare tire” exchange observed in other G4 structures with extra G-tracks [55–57]. Stochastic pUG folding is consistent with an equal distribution of 5′ and 3′ segments that can exchange into the fold, and the dynamic rearrangement of two out of the four segments of the pUG fold would explain the 50% amplitude of the fast phase. This hypothesis is also consistent with our data indicating expanded loops are tolerated in the pUG fold (Supplemental Fig. 10). Dynamic formation of loops is also consistent with the decrease in thermodynamic stability measured for longer pUGs (Supplemental Fig. 13). Loops in other G4 structures result in decreased thermodynamic stability [58]. If the fast exchange process involved other conformations (for example, duplexes or hairpins), then we would expect a length-dependence of the process, which is not observed. The hypothesis that the 5′ and 3′ segments are involved in the fast exchange dynamics is supported by the observation that the 3′ penultimate G23 imino has faster exchange dynamics than its symmetry related quartet partners (Fig. 6), and the observation that the 3′ terminal uridine is dynamic in solution [13] and disordered in the crystal [11]. In principle, the 50% fast phase kinetics may arise from a 50% signal change in all molecules or a 100% change in 50% of the molecules. However, the CD, NMR and native PAGE data all suggest that within the resolution of these methods, the molecules in this study adopt major conformations that can be assigned to the pUG fold or closely related variants such as those as depicted in Fig. 9. However, further investigations will be required to fully describe the dynamic conformational landscape of long pUG RNAs. Indeed, we observe the presence of additional peaks in the ^1^H NMR spectra of longer pUGs, as well as line broadening indicative of μs-ms exchange dynamics (Fig. 8). While a range of dynamic fluctuations were observed during the 3 μs MD simulation of (GU)_24_ (Fig. 5 and Supplemental data), further investigations are needed to determine the nature of μs-ms timescale dynamics in pUG RNAs.

We have previously shown that intermolecular right-handed pUG quadruplexes can form between short segments of 3-5 UG repeats (6-10 nt), but only at very high concentrations [48]. The dissociation constants for right-handed intermolecular pUG G4s range from 0.45 to 0.25 mM [48]. The linear trend in CD data (Fig. 2) and PAGE experiments (Fig. 4) show no evidence of right-handed intermolecular pUG G4s, which would not be expected at the low micromolar concentrations used in these experiments. Additionally, the NMR experiments were performed with RNA concentrations between 0.1 and 0.25 mM and the NMR data also show no evidence of right-handed intermolecular pUG G4s, as the observed chemical shifts correspond to the left-handed pUG fold. There is also no evidence for intermolecular associations of dimeric left-handed pUG folds [48] in the native PAGE data. The formation of dimeric pUG folds requires at least six unpaired repeats and thus far has only been observed for the short oligo (GU)_6_ [48]. We have previously shown that pUG fold formation of (GU)_12_ is concentration independent even at high concentrations [11]. In the case of longer pUGs such as (GU)_18_, we could envision that it is possible for both intramolecular and intermolecular pUG folds to occur simultaneously and cannot exclude this possibility. However, our data show that intramolecular pUG folds form stochastically within longer pUGs, which limits the availability of six unpaired repeats. Once the chain reaches 24 repeats, double intramolecular pUG folds are the major conformation.

Despite dynamic conformational exchange, the pUG fold core is stable for days. The kinetic stability of the pUG fold may provide a protective function in *C*. *elegans*, where pUG tails are RNA epigenetic modifications that are passed on for generations. The pUG fold must recruit RdRP to these templates, while protecting them from degradation by the 3′-5′ exoribonuclease MUT-7 which is also essential for gene silencing in *C*. *elegans* [59]. A proposed role of MUT-7 is to trim 3′ overhanging nucleotides from the pUG fold [59], which could lead to homogenous pUG folds by preventing the formation of loops. Kinetic stability may be important *in vivo*, as pUG fold-containing RNAs are packaged in embryos and inherited for generations in *C*. *elegans* [11, 20]. The pUG fold provides a unique, left-handed platform for proteins [39] and small molecule interactions [11]. Finally, thousands of potential pUG folds in humans may have important biological roles yet to be discovered.

## Supplementary Material

gkaf685_Supplemental_File

## Data Availability

The data underlying this article will be shared on reasonable request to the corresponding author.
